# A murine model of inflammation-induced cerebral microbleeds

**DOI:** 10.1186/s12974-016-0693-5

**Published:** 2016-08-30

**Authors:** Rachita K. Sumbria, Mher Mahoney Grigoryan, Vitaly Vasilevko, Tatiana B. Krasieva, Miriam Scadeng, Alexandra K. Dvornikova, Annlia Paganini-Hill, Ronald Kim, David H. Cribbs, Mark J. Fisher

**Affiliations:** 1 Department of Biopharmaceutical Sciences, School of Pharmacy, Keck Graduate Institute, Claremont, CA USA; 2 Department of Neurology, University of California, Irvine, CA USA; 3 Institute for Memory Impairments and Neurological Disorders, University of California, Irvine, CA USA; 4 Beckman Laser Institute, University of California, Irvine, CA USA; 5 Department of Radiology, University of California, San Diego, CA USA; 6 Department of Pathology and Laboratory Medicine, University of California, Irvine, CA USA; 7 Department of Anatomy and Neurobiology, University of California, Irvine, CA USA; 8 UC Irvine Medical Center, 101 The City Drive South, Shanbrom Hall, Room 121, Orange, CA 92868 USA

**Keywords:** Animal models, Cerebral microhemorrhage, Cerebral microbleeds, Inflammation, Hemosiderin

## Abstract

**Background:**

Cerebral microhemorrhages (CMH) are tiny deposits of blood degradation products in the brain and are pathological substrates of cerebral microbleeds. The existing CMH animal models are β-amyloid-, hypoxic brain injury-, or hypertension-induced. Recent evidence shows that CMH develop independently of hypoxic brain injury, hypertension, or amyloid deposition and CMH are associated with normal aging, sepsis, and neurodegenerative conditions. One common factor among the above pathologies is inflammation, and recent clinical studies show a link between systemic inflammation and CMH. Hence, we hypothesize that inflammation induces CMH development and thus, lipopolysaccharide (LPS)-induced CMH may be an appropriate model to study cerebral microbleeds.

**Methods:**

Adult C57BL/6 mice were injected with LPS (3 or 1 mg/kg, i.p.) or saline at 0, 6, and 24 h. At 2 or 7 days after the first injection, brains were harvested. Hematoxylin and eosin (H&E) and Prussian blue (PB) were used to stain fresh (acute) hemorrhages and hemosiderin (sub-acute) hemorrhages, respectively. Brain tissue ICAM-1, IgG, Iba1, and GFAP immunohistochemistry were used to examine endothelium activation, blood-brain barrier (BBB) disruption, and neuroinflammation. MRI and fluorescence microscopy were used to further confirm CMH development in this model.

**Results:**

LPS-treated mice developed H&E-positive (at 2 days) and PB-positive (at 7 days) CMH. No surface and negligible H&E-positive CMH were observed in saline-treated mice (*n* = 12). LPS (3 mg/kg; *n* = 10) produced significantly higher number, size, and area of H&E-positive CMH at 2 days. LPS (1 mg/kg; *n* = 9) produced robust development of PB-positive CMH at 7 days, with significantly higher number and area compared with saline (*n* = 9)-treated mice. CMH showed the highest distribution in the cerebellum followed by the sub-cortex and cortex. LPS-induced CMH were predominantly adjacent to cerebral capillaries, and CMH load was associated with indices of brain endothelium activation, BBB disruption, and neuroinflammation. Fluorescence microscopy confirmed the extravasation of red blood cells into the brain parenchyma, and MRI demonstrated the presence of cerebral microbleeds.

**Conclusions:**

LPS produced rapid and robust development of H&E-positive (at 2 days) and PB-positive (at 7 days) CMH. The ease of development of both H&E- and PB-positive CMH makes the LPS-induced mouse model suitable to study inflammation-induced CMH.

## Background

Cerebral microhemorrhages (CMH) are tiny perivascular deposits of blood degradation products in the brain and are the pathological substrate of cerebral microbleeds [1]. In spite of the significant clinical and scientific interest in this field, lack of appropriate animal models has hindered progress in delineating the exact mechanisms involved in CMH development and in the development of treatments to address CMH. The currently used animal models of CMH are amyloid beta (Aβ)- [2–4], hypoxia-reoxygenation-, or hypertension-induced [5]. These existing animal models have several disadvantages: (1) CMH development in these models can take up to 15–24 months, (2) invasive surgical procedures are required to exacerbate CMH development, and most importantly, (3) clinically, CMH may develop independent of amyloid deposition, hypoxic brain injury, or hypertension [6].

It is now well recognized that CMH are not only associated with cerebrovascular diseases including stroke, cerebral amyloid angiopathy (CAA), and cerebral hypertensive vasculopathy [1] but are also found in patients with sepsis [7], Parkinson’s disease [8], chronic obstructive pulmonary disease (COPD) [9], and traumatic brain injury (TBI) [10] and in normal aging adults [1]. One common feature of these entities is systemic inflammation [11], and recent human studies show a link between systemic inflammation and CMH pathogenesis. Higher levels of peripheral inflammatory markers are associated with cerebral microbleeds in aging patients [12], high levels of circulating tumor necrosis factor receptor 2 are observed in subjects with cerebral microbleeds [13], and higher activity of lipoprotein phospholipase-A2 (a marker of vascular inflammation) is related to the presence of deep cerebral microbleeds in subjects who were carriers of at least one APOE ε2 or ε4 allele [14]. While inflammation may be central to the development of CMH, a direct causal link between inflammation and CMH development has been lacking. Hence, we hypothesized that systemic inflammation will induce CMH development and that an inflammation-induced animal model will be appropriate to study CMH development and treatment.

To test this hypothesis, we used lipopolysaccharide (LPS), a well-characterized standardized inflammatory stimulus, to study CMH development. Previous studies from our lab showed that mice treated with a 5 mg/kg dose of LPS at 0 and 24 h had a significantly higher number of CMH at 2 days as evident by hematoxylin and eosin (H&E)-positive staining in brain tissue compared with controls. We now report a well-characterized inflammation-induced mouse model of CMH with low mortality, using different dosing regimens (1 or 3 mg/kg, i.p., at 0, 6, and 24 h, and sacrifice at 2 or 7 days) of LPS. In the current study, we examined both acute CMH (H&E-positive) at 2 days and sub-acute CMH (Prussian blue/hemosiderin-positive) at 7 days. The number, size, total area, and neuroanatomical distribution of CMH were examined in the LPS- and saline-treated mice. To elucidate mechanisms involved in LPS-induced CMH, we analyzed markers of brain endothelial damage (ICAM-1 and parenchymal IgG) and neuroinflammation (astrocyte and microglia/macrophages). The vascular source of LPS-induced CMH was examined, and we also used fluorescence microscopy to confirm acute CMH development and magnetic resonance imaging (MRI) to confirm the radiographic presence of cerebral microbleeds.

## Methods

### Mouse treatment

All animal procedures were approved by the UCI Institutional Animal Care and Use Committee and were carried out in compliance with the University Laboratory Animal Resources regulations. Adult (male and female 10–12 weeks old) C57BL/6 mice (Taconic, Hudson, NY) were used for all the experiments. In the first set of experiments, the mice were treated with either a 3 mg/kg dose of LPS derived from *Salmonella typhimurium* (Sigma, St. Louis, MO) or saline i.p. at 0, 6, and 24 h and sacrificed 2 days after the first injection to examine acute CMH development. In a separate series of experiments, the mice were treated with a 1 mg/kg dose of LPS or saline at 0, 6, and 24 h and sacrificed 7 days after the first injection to examine sub-acute CMH development. The mice fed and drank ad lib and received up to three times daily doses of 1 cm^3^ saline subcutaneously. Two or 7 days after the first injection, mice were anesthetized with a lethal dose of Nembutal (150 mg/kg, i.p.), cardiac perfusions were performed using ice-cold PBS for 5 min to clear the cerebral vasculature, and brains were processed for CMH detection.

### Microhemorrhage detection

Brains were fixed in 4 % paraformaldehyde at 4 °C for 72 h, examined for surface microhemorrhages, and sectioned into 40-μm coronal sections using a vibratome (Technical Products International, Inc., St. Louis, MO). Every sixth section was collected and stained with either H&E to detect fresh (acute) microhemorrhages in the 2-day study or Prussian blue (PB) to detect hemosiderin (a marker of sub-acute microhemorrhage) in the 7-day study. PB was not used for the 2-day study, based on findings of our earlier work [15]. A total of approximately 30 brain sections were analyzed per mouse. For PB staining, sections were stained using freshly made 5 % potassium hexacyanoferrate trihydrate (Sigma, St. Louis, MO) and 5 % hydrochloric acid (Sigma, St. Louis, MO) for 30 min, rinsed in water and counterstained with Nuclear Fast Red (Sigma, St. Louis, MO), dehydrated, and cover-slipped. H&E staining was performed by Research Services Core offered by the Department of Pathology and Laboratory Medicine at the UCI Medical Center. CMH were counted at a ×20 magnification by a blinded observer as a collection of red blood cells (RBC) that appear red-orange using H&E stain and as clear purple-blue deposits using PB. Digitized images were obtained using an Olympus BX40 microscope and CC-12 Soft-Imaging System with Olympus MicroSuite (TM)-B3SV software. CMH size (μm^2^) and positive area (expressed as a percent of total area analyzed) were determined by an observer blinded to the experiment using the NIH ImageJ software 1.62. When the vessel associated with the CMH was visible, internal diameter of the blood vessel was determined using the NIH ImageJ software 1.62.

### ICAM-1, IgG, Iba1, and GFAP immunohistochemistry

To determine the role of endothelial damage and neuroinflammation in LPS-induced CMH development, ICAM-1 (marker of endothelial cell activation), parenchymal IgG (blood-brain barrier (BBB) damage marker), Iba1 (microglia/macrophage marker), and GFAP (astrocyte marker) immunohistochemistry were performed. Briefly, 40-μm sections from mice treated with LPS and saline from the 2-day study were incubated in 0.5 % hydrogen peroxide in 0.1 M PBS (pH 7.4) containing 0.3 % Triton-X100 (PBST) for 30 min at room temperature to block endogenous peroxidase activity. After washing with PBST, sections were incubated for 30 min with PBST containing 2 % bovine serum albumin to block non-specific protein binding. Sections were then incubated overnight at 4 °C with a rabbit anti-mouse IgG antibody (1:200 dilution; Jackson ImmunoResearch, West Grove, PA), rabbit monoclonal antibody against ICAM-1 (1:500 dilution; Abcam, Cambridge, MA), rabbit antibody against Iba1 (1:200 dilution, Wako Chemicals USA, Richmond, VA), or rabbit antibody against GFAP (1:2000 dilution, Abcam, Cambridge, MA). After washing with PBST, sections were incubated at room temperature for 1 h with biotinylated anti-rabbit IgG (1:500 dilution; Jackson ImmunoResearch, West Grove, PA), followed by 1-h incubation at room temperature with ABC complex according to the manufacturer instructions (Vector Laboratories, Burlingame, CA). Sections were developed with 3,3′-diaminobenzidine (DAB) (Vector Laboratories, Burlingame, CA). Sixteen images per brain section were acquired at ×20 magnification, and the total positive immunoreactive area was quantified using the NIH ImageJ software by an observer blinded to the experimental groups. Immunopositive area was expressed as percent of total area analyzed.

### Magnetic resonance imaging

MRI was performed to confirm the radiographic presence of cerebral microbleeds in this model using a randomly selected subset of mouse brains after completion of the 7-day experiment. Mouse brains were collected post mortem after cardiac perfusion with PBS to clear vasculature of blood, followed by fixation in 4 % paraformaldehyde; imaging was performed prior to sectioning. Data were acquired using a Bruker 7T small-animal MRI machine with a 12-cm gradient, a 660 m/T/m strength and 4570 slew rate, and a 1-cm receive-only surface coil. The pulse sequence used was a 3D FLASH (fast low-angle shot) gradient echo sequence. TE was 12 ms, TR was 30.2 ms, and FA was 11°. Voxel size was 100 × 100 × 156 μm. The MRI data was manually processed and surface-rendered using Amira software (FEI, Hillsboro, OR).

### Ex vivo optical microscopy imaging

In a separate series of experiments, the transit of RBC across the cerebral vasculature into the brain parenchyma was visualized using ex vivo confocal and two-photon-excited fluorescence (TPEF) microscopy. Briefly, autologous blood was collected from inbred Tie2-GFP mice (Jackson Laboratory, Bar Harbor, ME) in which endothelial cells are labeled with green fluorescent protein (GFP), and RBC were purified using Ficoll-Paque (GE Healthcare, Uppsala, Sweden) gradient. After several washes in PBS, RBC were stained with PKH26 Red Fluorescent Cell Linker Kit (Sigma, St. Louis, MO) according to the manufacturer’s instructions and re-injected into the mice. Immediately after RBC injection, the mice were subjected to either saline or LPS triple-dosing regimen (3 mg/kg, i.p., 0, 6, and 24 h) as per the 2-day experiment described earlier. The mice were sacrificed 48 h after the first saline or LPS injection, and whole brains were collected and fixed in 4 % paraformaldehyde for imaging. Fluorescence and second-harmonic generation (SHG) images of whole brains fixed in 4 % paraformaldehyde were obtained using Zeiss LSM 510 Meta NLO microscopy system equipped with a long working distance Zeiss 40 × 0.8 water immersion objective. GFP fluorescence was excited by 488-nm line of the Argon laser; PKH26 excitation was provided by He-Ne 543-nm laser, and SHG signal from collagen was generated using a Chameleon Ultra femtosecond pulsed tunable laser (Coherent Inc., Los Angeles, CA) at 800 nm. Two confocal fluorescence channels (green emission at 500–530 nm and red emission at 565–615 nm) were acquired simultaneously, and SHG image (blue emission filter 390–465 nm) was acquired consequently. Laser scanning did not induce any visible damage to the cells or noticeable bleaching of the sample. Stacks of images were acquired with the *z*-step (distance between consecutive imaging planes) of 2.5 μm. The maximum depth for imaging was up to 80 μm from the brain surface. The probed 3D volume was reconstructed by Zeiss LSM original software.

### Statistical analysis

Data were represented as mean ± SEM, and all statistical analysis was performed using GraphPad Prism 5 (GraphPad Software Inc., La Jolla, CA). Student’s *t* test (for normally distributed data) or Mann-Whitney *U* test (for non-normal data) was used to compare two groups. One- and two-way ANOVA (with and without repeated measures) with Bonferroni’s post hoc test were used to compare more than two groups. Spearman’s rho correlation was used for correlation analysis. A *p* value of <0.05 was considered statistically significant.

## Results

### Survival

For the 2-day experiment, all 12 mice in the saline group survived the duration of the study; however, one mouse in the LPS group died before the end of the study, and two were excluded due to incomplete perfusion, giving us a total of ten mice in the LPS group. All mice (*n* = 9 per group) survived the 7-day experiment.

### Surface cerebral microhemorrhages

**Fig. 1:**
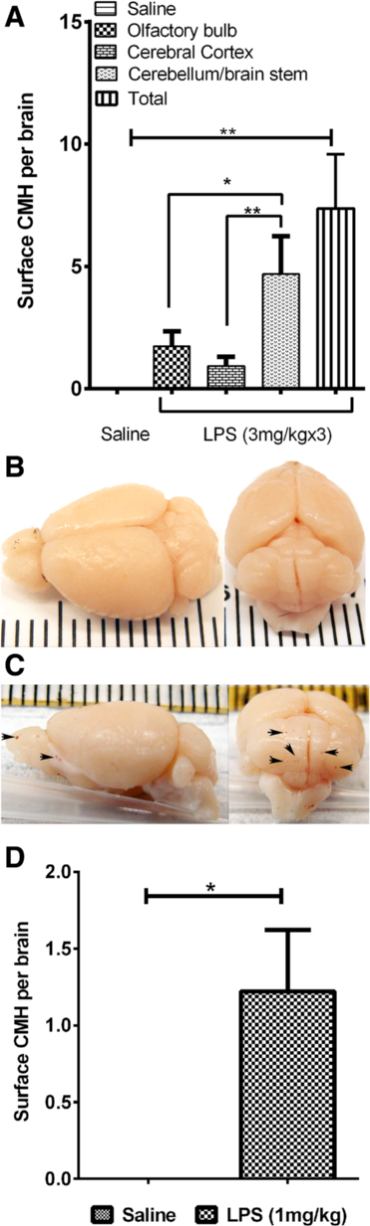
Significant development of surface microhemorrhages in mice treated with LPS (*n* = 10) (3 mg/kg at 0, 6, and 24 h) compared with saline-treated mice (*n* = 12) and regional differences in the distribution of surface microhemorrhages in LPS-treated mice (**a**) in the 2-day experiment. Brain images showing absence of surface microhemorrhages in saline-treated mice (**b**) and presence of surface microhemorrhages in the olfactory bulb (*left panel*) and the cerebellum (*right panel*) of LPS-treated mice (**c**). Significantly higher surface microhemorrhages in mice treated with LPS (*n* = 9) (1 mg/kg at 0, 6, and 24 h) compared with the saline-treated mice (*n* = 9) in the 7-day experiment (**d**). Data are presented as mean ± SEM. Student’s *t* test for two groups and one-way ANOVA with Bonferroni’s post-test for more than two groups; *p < 0.05, **p < 0.01

In the 2-day experiment, a 3 mg/kg dose of LPS at 0, 6, and 24 h induced the development of grossly visible surface CMH (7.4 ± 2.2 per brain), compared with saline controls that exhibited no surface CMH (*p* < 0.01) (Fig. 1a–c). Regional differences were observed in the location of these surface CMH. In the LPS-treated group, the average number of surface CMH per brain was significantly higher in the cerebellum/brain stem (4.7 ± 1.6) compared with the olfactory bulb (1.8 ± 0.6, *p* < 0.05) and the cerebral cortex (0.9 ± 0.3, *p* < 0.01) (Fig. 1a). In mice treated with a 1 mg/kg dose of LPS at 0, 6, and 24 h and sacrificed 7 days after the first injection, the average number of surface CMH (1.2 ± 0.4 per brain) was significantly higher compared with the saline-treated mice, which again exhibited no surface CMH (*p* < 0.05; Fig. 1d).

### Acute parenchymal cerebral microhemorrhages

**Fig. 2:**
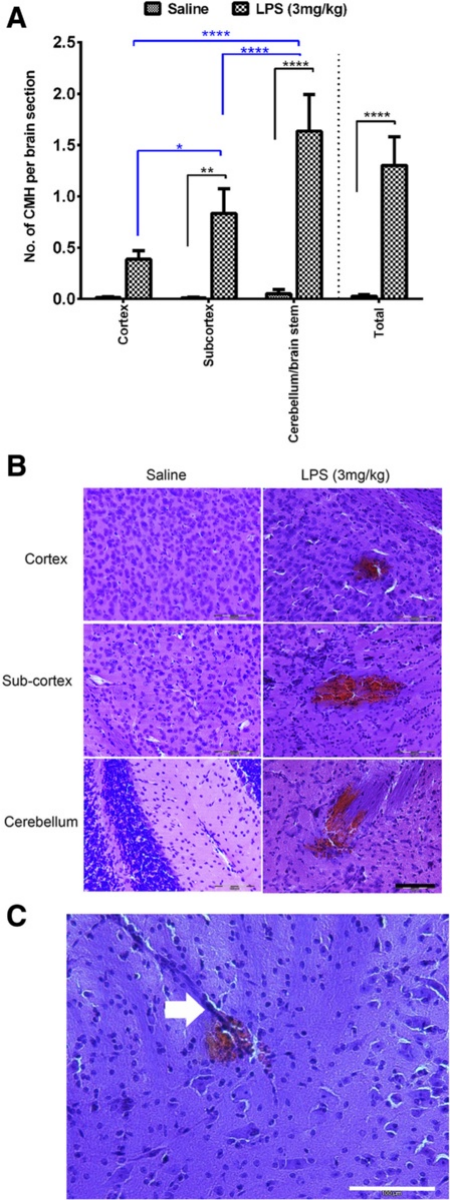
Acute H&E-positive cerebral microhemorrhages: Significant development of acute (H&E-stained) parenchymal microhemorrhages in mice treated with LPS (*n* = 10) (3 mg/kg, at 0, 6, and 24 h) compared with saline-treated mice (*n* = 12) (comparisons shown in *black*) (**a**). Significant difference in regional distribution of H&E-positive parenchymal microhemorrhages in the LPS-treated group (comparisons shown in *blue*) (**a**). Representative images showing H&E-positive cerebral microhemorrhages in the cortex, sub-cortex, and cerebellum of LPS- and saline-treated mice (**b**). Representative image showing a cerebral blood vessel associated with an acute (H&E-positive) cerebral microhemorrhage (**c**). Data are presented as mean ± SEM. Two-way repeated measure ANOVA with Bonferroni’s post-test; *p < 0.05, **p<0.01, ****p < 0.0001. Scale bar = 100 μm

In the 2-day experiment, a 3 mg/kg dose of LPS at 0, 6, and 24 h significantly induced (*p* < 0.0001 vs saline control) the formation of fresh H&E-positive parenchymal CMH (1.3 ± 0.3 vs 0.03 ± 0.02 per brain section). H&E-positive CMH were observed in all LPS-treated mice (ranging from 0.4 to 2.9 per brain section). Regional differences were observed in the distribution of H&E-positive CMH in the LPS-treated mice (Fig. 2a, b). The number of H&E-positive CMH was the highest in the cerebellum/brain stem (1.6 ± 0.4 per brain section) followed by the sub-cortical (0.8 ± 0.2 per brain section, *p* < 0.0001 vs cerebellum and *p* < 0.05 vs cortex) and the cortical (0.4 ± 0.09 per brain section, *p* < 0.0001 vs cerebellum) regions of the brains of LPS-treated mice. No regional differences were observed in the distribution of H&E-positive CMH in the saline-treated mice (cortex 0.01 ± 0.007, sub-cortex 0.01 ± 0.007, and cerebellum/brain stem 0.05 ± 0.04 per brain section). H&E-positive CMH were also examined in the 7-day study to see if acute CMH continued to develop after LPS injections were stopped at 2 days. No significant differences in the number of H&E-positive CMH were found between the LPS- and saline-treated mice in the 7-day study (data not shown).

A total of 68 blood vessels associated with H&E-positive CMH were visible and studied in the current study. Of these, 51 were <10 μm in diameter (mean diameter 5.6 ± 0.04 μm) and 17 were between 10 and 30 μm in diameter (mean diameter 15.8 ± 1.7 μm). The mean diameter of the vessels associated with the H&E-positive CMH was 8.2 ± 0.09 μm (Fig. 2c), and the blood vessels ranged from 1.5 to 30 μm in diameter.

**Fig. 3:**
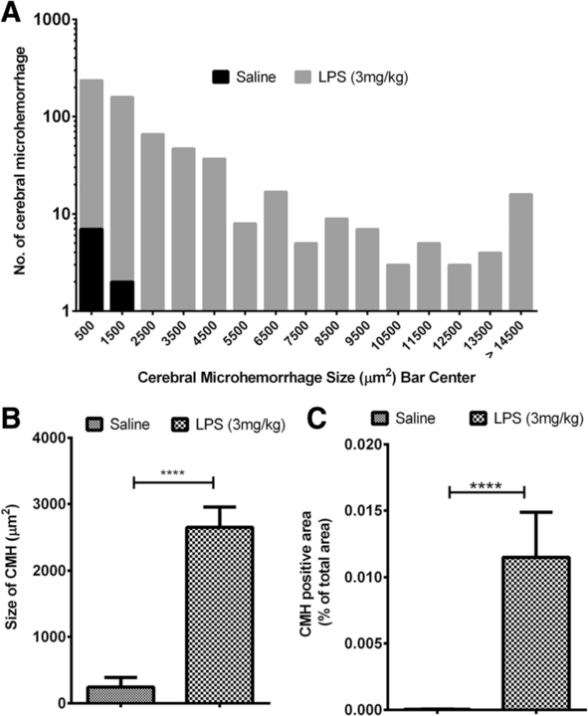
Size and area of acute H&E-positive CMH: Size distribution of all the H&E-positive CMH analyzed in the LPS (*n* = 10) (3 mg/kg, at 0, 6, and 24 h)- and saline (*n* = 12)-treated mice (**a**). The width of each bar is 1000 μm^2^, and the bin center for each bar is shown on the *X*-axis. H&E-stained cerebral microhemorrhage average size (**b**) and positive area (**c**) are significantly higher in LPS-treated (3 mg/kg, at 0, 6, and 24 h) mice compared with saline controls. Data are presented as mean ± SEM. Mann-Whitney *U* test to compare two groups; ****p < 0.0001

Approximately 30 sections per mouse brain were examined in the current study. To study the size distribution of the parenchymal CMH, all observed CMH from all the mice in each group were studied. A total of 616 (from *n* = 10) and 9 (from *n* = 12) H&E-positive CMH were analyzed in the LPS- and saline-treated groups, respectively. H&E-positive CMH ranged from 136 to 1684 μm^2^ in the saline group and from 63 to 3.9 × 10^6^ μm^2^ in the LPS group (Fig. 3a). LPS produced H&E-positive CMH that were significantly larger in size compared with saline controls (*p* < 0.0001). The mean size of H&E-positive CMH was 2649 ± 306 and 246 ± 145 μm^2^ in the LPS- and saline-treated mice, respectively (Fig. 3b). Total H&E-positive CMH area (or total CMH load, is a function of both the number and size of microhemorrhages) was significantly higher (*p* < 0.0001) in the LPS- compared with the saline-treated group (0.01± 0.004 % vs 4.8 x 10^-5 ^ ± 2.7 x 10^-5^ % of total brain section analyzed area) (Fig. 3c). The weights of the mice at the beginning and end of the 2-day study were 21.8 ± 0.9 and 21.3 ± 0.8 g in the LPS group and 21.9 ± 0.7 and 22.6 ± 0.76 g in the saline group, respectively.

### Sub-acute parenchymal cerebral microhemorrhages

**Fig. 4:**
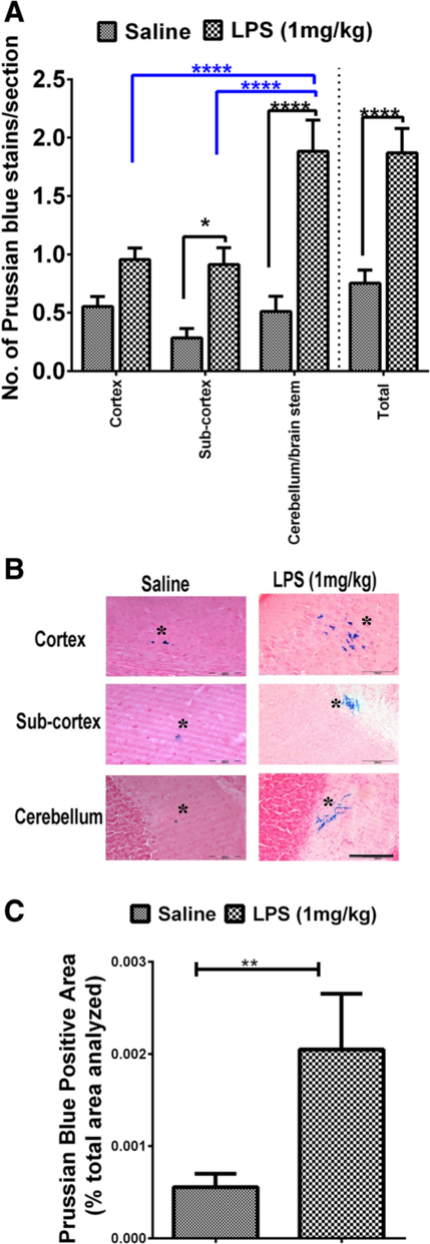
Sub-acute Prussian blue-positive cerebral microhemorrhages: Significantly higher number of PB-positive stains in LPS (1 mg/kg, at 0, 6, and 24 h; *n*=9)- compared with saline-treated (*n*=9) mice (comparisons shown in *black*) (**a**). Differences in regional distribution of PB-positive stains in the cortex, sub-cortex, and cerebellum of LPS- and saline-treated mice (comparisons shown in *blue*) (**a**). Representative images showing PB-positive stains in different brain regions (**b**). Scale bar = 100 μm. Significantly higher PB-positive area in the LPS- compared with saline-treated mice (**c**). Data are presented as mean ± SEM. Mann-Whitney *U* test to compare two groups and two-way repeated measure ANOVA with Bonferroni’s post-test for more than two groups; *p < 0.05, **p < 0.01, ****p < 0.0001

PB-positive stains were detected in both mice treated with saline and 1 mg/kg dose of LPS at 0, 6, and 24 h and sacrificed at 7 days. However, the mean number of PB-positive stains was significantly higher (*p* < 0.0001) in the LPS-treated group (1.9 ± 0.2 per brain section) compared with saline control (0.8 ± 0.1 per brain section) (Fig. 4a). Similar to H&E-positive fresh CMH, PB-positive stains showed differences in regional distribution among the LPS-treated mice (Fig. 4b, c). The number of PB-positive stains was significantly higher in the cerebellum/brain stem (1.9 ± 0.3 per brain section) compared with the cortical (1.0 ± 0.1 per brain section, *p* < 0.0001) and the sub-cortical (0.9 ± 0.1 per brain section, *p* < 0.0001) regions of the brains of LPS-treated mice. No regional differences were observed in the distribution of PB-positive stains in the saline-treated mice (cortex 0.6 ± 0.09, sub-cortex 0.3 ± 0.08, and cerebellum/brain stem 0.5 ± 0.1 per brain section). A total of 466 (from *n* = 9) and 231 (from *n* = 9) PB-positive stains were analyzed in the LPS- and saline-treated groups, respectively. PB-positive stains ranged from 9 to 29,377 μm^2^ among the LPS mice and from 14 to 4888 μm^2^ among the saline-treated mice. The average PB-positive stain size was not significantly different in the LPS- and saline-treated mice (data not shown). The total PB-positive area was significantly (*p* < 0.01) higher in the LPS-treated mice (0.002 ± 0.0006 % of total brain section area analyzed) compared with saline controls (0.0006 ± 0.0001 % of total brain section area analyzed) (Fig. 4c). For the 7-day study, the average weights of the mice at the beginning and end of the study were 21.0 ± 0.6 and 22.1 ± 0.7 g among the saline mice and 20.7 ± 1 and 20.6 ± 0.7 g among the LPS mice.

### Correlation between CMH development and brain endothelial damage

Immunohistochemical analysis using sections (eight mice from the LPS-treated group and four mice from the saline-treated group) from the 2-day experiment revealed that ICAM-1-positive area was significantly associated with the number of CMH per brain section (*r* = 0.86, *p* < 0.001) and total CMH-positive area (or CMH load) expressed as % total area (*r* = 0.70, *p* < 0.01). IgG-positive area was significantly associated with CMH size (*r* = 0.56, *p* < 0.05) and total CMH-positive area (or CMH load) (*r* = 0.53, *p* < 0.05), as shown in Table 1.

**Table 1:** Correlation between ICAM-1-, IgG-, Iba1-, and GFAP-positive area with the (a) number of CMH, (b) CMH size, and (c) CMH-positive area in the 2-day experiment

	ICAM-1-positive area (%) *r*	IgG-positive area (%) *r*	Iba1-positive area (%) *r*	GFAP-positive area (%) *r*
No. of CMH/section	0.86***	0.38	0.85***	0.83**
CMH size (μm^2^)	0.31	0.56*	0.72**	0.62*
CMH-positive area (% total area)	0.70**	0.53*	0.88***	0.85***

One-tailed *p* values: *p < 0.05, **p < 0.01, ***p < 0.001

### Correlation between CMH development and neuroinflammation

**Fig. 5:**
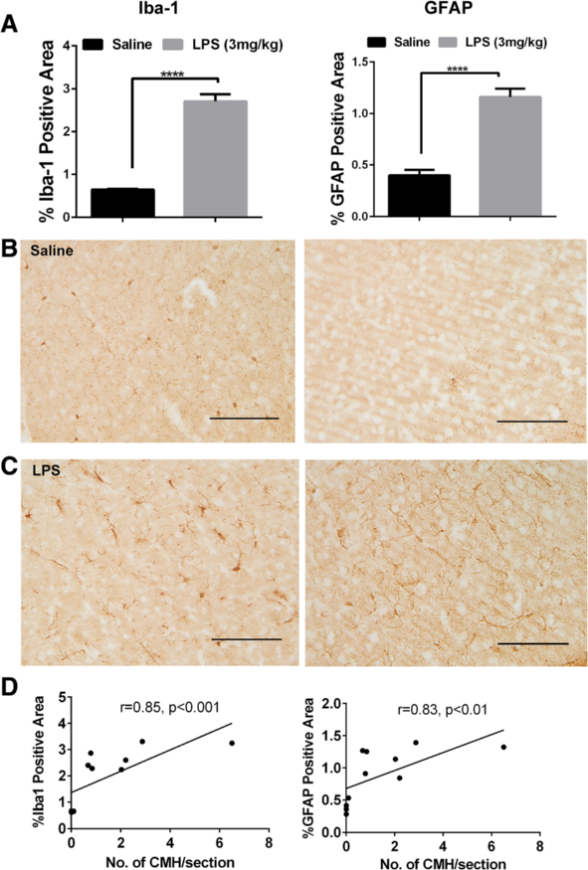
Neuroinflammation and CMH development: Significantly higher Iba1 (*left panel*)- and GFAP (*right panel*)-positive immunoreactive area in the LPS-treated mice (*n* = 7) (3 mg/kg, at 0, 6, and 24 h) compared with saline controls (*n* = 4) (**a**) in the 2-day model. Representative images showing Iba1 (*left*)- and GFAP (*right*)-positive immunoreactive area in the saline (**b**)- and LPS (**c**)-treated mice (scale bar = 50 μm; images were taken at ×20). The CMH number was significantly associated with both Iba1 (*left*)- and GFAP (*right*)-positive immunoreactive area in the 2-day model (3 mg/kg, at 0, 6, and 24 h) (**d**). Data are presented as mean ± SEM. Student’s *t* test to compare two groups; ****p < 0.0001; one-tailed Spearman rho correlation for correlation analysis

Immunohistochemical analysis using brain sections (seven mice from the LPS-treated group and four mice from the saline-treated group) from the 2-day experiment showed that the total Iba1- and GFAP (markers of neuroinflammation)-positive immunoreactive areas were significantly higher in the LPS-treated mice compared with the saline controls (Fig. 5a–c). Further, both Iba1- and GFAP-positive immunoreactive areas were significantly associated with the number (Iba1 *r* = 0.85, *p* < 0.01; GFAP *r* = 0.83, *p* < 0.01; Fig. 5d), size (Iba1 *r* = 0.72, *p* < 0.01; GFAP *r* = 0.62, *p* < 0.05), and total CMH load (Iba1 *r* = 0.88, *p* < 0.001; GFAP *r* = 0.85, *p* < 0.001), as shown in Table 1.

**Fig. 6:**
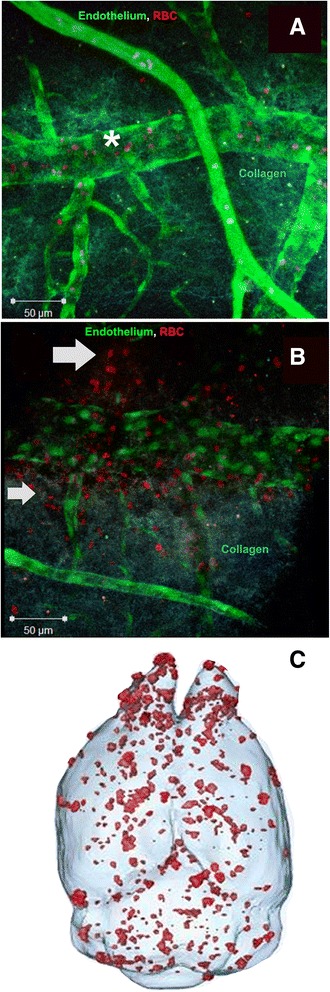
Ex vivo fluorescence and second-harmonic generation imaging showing SHG of collagen (*blue color*), fluorescence of GFP in endothelial cells (*green*), and PKH26 fluorescence marker in red blood cells (*red*) in the brains of saline (**a**) and triple-dose (3 mg/kg at 0, 6, and 24 h) LPS-treated Tie2-GFP mice sacrificed at 2 days (**b**). All images are 3D reconstructions in enface (or true focus) geometry. *Asterisk sign* indicates RBC confined within the brain vasculature in a saline mouse (**a**), and *arrows* indicate extravasation of RBC from the brain vasculature into the brain parenchyma in a LPS-treated mouse (**b**). Superior perspective of 3D surface renderings of brain showing distribution of cerebral microbleeds (in *red*) in mice from the 7-day experiment treated with a triple-dosing regimen of LPS (1 mg/kg at 0, 6, and 24 h) (**c**) using T2*-weighted 3D FLASH MRI

Apart from histochemistry, in the current model, the development of parenchymal CMH and cerebral microbleeds was further confirmed by two separate methodologies. In the 2-day experiment, ex vivo confocal fluorescence microscopy showed the transit of fluorescently labeled RBC (PKH26 labeled red RBC; Fig. 6a, b) across the cerebral vascular endothelium (GFP labeled green endothelium; Fig. 6a, b) into the brain parenchyma in the LPS-treated mice (Fig. 6b) but not the saline-treated mice (Fig. 6a). In the 7-day experiment, MRI confirmed robust development of cerebral microbleeds in the LPS-treated mice (Fig. 6c).

## Discussion

In the current study, we have developed and characterized an LPS-induced inflammation mouse model of CMH that shows rapid and robust development of CMH. As early as 2 days following an intraperitoneal LPS triple-dosing regimen (3 mg/kg at 0, 6, and 24 h), H&E-positive (acute) CMH were observed in mice. PB-positive CMH were observed in mice at 7 days following a modified intraperitoneal LPS triple-dosing regimen (1 mg/kg at 0, 6, and 24 h). Both the H&E- and PB-positive lesions showed similar neuroanatomical distribution patterns, with the highest number of CMH located in the cerebellum followed by the sub-cortex and cortex. The total CMH load was significantly associated with markers of cellular activation (endothelium, astrocyte, and microglia/macrophage) and BBB disruption. Finally, MRI confirmed the presence of cerebral microbleeds in this LPS-induced inflammation mouse model.

Systemic inflammation and an increase in inflammatory markers have been associated with normal aging [11], stroke [16–18], ﻿﻿and peripheral conditions including sepsis [19] and hypertension [20]. Active inflammation can cause endothelial damage and BBB disruption [21], and it is speculated that these events allow extravasation of erythrocytes from the blood vessel lumen into the brain parenchyma, resulting in CMH development [22]. In fact, recent studies show an association between active inflammation and CMH [12, 13]. Inflammation may thus produce vascular dysfunction that triggers or potentiates CMH development.

LPS is a well-studied inflammatory stimuli used in rodent models of acute inflammation [23] that causes BBB damage [24] and brain endothelial dysfunction [25, 26]. These characteristics of LPS make it a well-suited stimulus for the development of inflammation-induced CMH. We have previously shown that two intraperitoneal injections of LPS (5 mg/kg at 0 and 24 h) result in the development of H&E-positive CMH in C57BL/6 mice, at 2 days after the first LPS injection [15]. In the current study, we used a modified dosing regimen, adapted from a published study [27] and found that triple intraperitoneal injections of LPS (3 mg/kg at 0, 6, and 24 h) result in rapid (as early as 2 days) development of H&E-positive CMH, with negligible mortality.

CMH are primarily found to be associated with a rupture in the cerebral vessel wall that allows extravasation of RBC into the brain parenchyma [28]. In our model, LPS-induced CMH were significantly associated with endothelial activation (determined by measuring ICAM-1 immunoreactivity) and BBB disruption (determined by measuring brain IgG immunoreactivity). Further, larger CMH were associated with a larger BBB disruption. LPS can trigger an inflammatory response within the brain parenchyma, and studies show that neuroinflammation is associated with microvascular injury and microhemorrhage development [29, 30]. In the present study, endothelial damage along with neuroinflammation (evident from the increase in microglia/macrophage- and astrocyte-positive immunoreactivity in LPS-treated mice) were significantly associated with CMH development, indicating a role for BBB injury and neuroinflammation in the pathogenesis of LPS-induced CMH.

In the current study, LPS-induced CMH developed at the level of both the cerebral capillaries (vessel diameter <10 μm) [31] and larger vessels (vessel diameter >10 μm) [32].The majority of the vessels associated with LPS-induced CMH were <10 μm in diameter, suggesting that brain capillaries [31] are more susceptible to LPS-induced CMH development in this model. Our finding of a capillary involvement in CMH development is consistent with recent rodent studies in which hypertension- and hypoxia-induced CMH were also localized around cerebral microvessels [5, 33].

Existing mouse models of CMH include mice that over-express mutant amyloid precursor protein to study cerebral amyloid angiopathy (CAA)-associated CMH [2], hypertensive mice [5] to study hypertension-associated CMH, and the hypoxia-reoxygenation-induced CMH mouse model to study high-altitude-associated CMH [33]. In the CAA mouse model, mice spontaneously develop PB-positive lesions by 15–24 months of age [2] and development of CMH can be exacerbated by passive immunization [3, 34] or by inducing bilateral common carotid artery stenosis [4]. Two-photon-excited microscopy has been used to induce localized cortical CMH [35], and hyperhomocysteinemia (HHcy) has also been used to produce CMH [36]. One commonality between the existing mouse models of CMH and the present LPS-induced mouse model is inflammation. CMH development was found to be associated with an enhanced M1-type neuroinflammatory response in the CAA mouse model, with increased microglial activation and brain cytokine levels in the HHcy mouse model, and a rapid inflammatory response up to 200 μm from CMH in the two-photon-excited microscopy mouse model. These observations are consistent with the current model where we observed a significant association between markers of neuroinflammation (microglia/macrophage and astrocytes) and CMH development.

In the existing rodent models [2, 3, 34, 36] and clinically [6], PB staining for hemosiderin is commonly used to detect old CMH in post mortem brain tissues. Further, the current standardized method used to visualize cerebral microbleeds in humans is MRI, which relies on the paramagnetic properties of hemosiderin. Detection of hemosiderin in brain tissue is thus crucial to develop a clinically relevant mouse model of cerebral microbleeds. PB-positive lesions cannot be detected 2 days after LPS injection [15], and we thus modified the dosing regimen and extended our study to 7 days to detect PB-positive lesions and cerebral microbleeds using MRI in our model. C57BL/6 mice treated with a triple intraperitoneal dose of LPS (1 mg/kg at 0, 6, and 24 h) showed robust development of PB-positive lesions at 7 days with no mortality. Visualization of LPS-induced CMH at 7 days using MRI demonstrates and confirms radiologically the presence of cerebral microbleeds in the current model.

Clinically, typical CMH location is associated with distinct underlying vascular pathologies: hypertensive vasculopathy is associated with CMH that are located in the basal ganglia, thalamus, cerebellum, and brain stem, while CAA is associated with lobar CMH [1]. Varied neuroanatomical CMH distribution is also observed in hypoxia-reoxygenation- and hypertension-induced CMH mouse models; however, CMH density in these models is higher in the cortex or the olfactory bulb [5, 33]. In the current LPS model, we saw widespread distribution of both H&E- and PB-positive lesions. The regional distribution patterns of both H&E- and PB-positive lesions were similar with maximum CMH density in the cerebellum and the brain stem region followed by the cerebrum (sub-cortex and cortex). Surface CMH that were grossly visible on the brain surface post-LPS treatment were also significantly higher in the cerebellum and brain stem region. The presence of surface CMH suggests a phenomenon similar to cortical superficial siderosis [37], and more extensive analysis of these lesions by MRI will clarify whether the processes are fundamentally similar. Though the mechanisms underlying the observed regional distribution pattern of CMH in the current study are not clear, higher distribution in the cerebellum most likely relates to the vulnerability of cerebellum blood vessels to inflammation.

## Conclusions

Compared to the existing mouse models of CMH, the current model appears to offer considerable advantages. The LPS-induced mouse model is inflammation-based (not limited to CAA-, hypoxic brain injury-, or hypertension-induced CMH), non-invasive, and shows rapid (2 and 7 days) development of CMH with negligible mortality. Our work shows that LPS-induced CMH are primarily associated with cerebral capillaries, and BBB dysfunction and neuroinflammation play a role in CMH development in this model. Additionally, the cerebellum/brain stem region is more susceptible to LPS-induced CMH development. The ease of development of both H&E- and PB-positive CMH, and MRI-visible cerebral microbleeds makes the LPS-induced mouse model suitable to study the pathophysiology of inflammation-induced CMH.

## Abbreviations

CMH: Cerebral microhemorrhages
LPS: Lipopolysaccharide
H&E: Hematoxylin and eosin
PB: Prussian blue
MRI: Magnetic resonance imaging
CAA: Cerebral amyloid angiopathy
COPD: Chronic obstructive pulmonary disease
TBI: Traumatic brain injury
DAB: 3,3′-Diaminobenzidine
TPEF: Two-photon-excited fluorescence
BBB: Blood-brain barrier
